# CUBIC: A Versatile Cumate-Based Inducible CRISPRi System in *Streptomyces*

**DOI:** 10.1021/acssynbio.3c00464

**Published:** 2023-10-06

**Authors:** Chaoxian Bai, Gilles P. van Wezel

**Affiliations:** Institute of Biology, Leiden University, Sylviusweg 72, 2333 BE, Leiden, Netherlands

**Keywords:** *Streptomyces*, cumate inducible promoter, CRISPR interference, natural products, synthetic biology

## Abstract

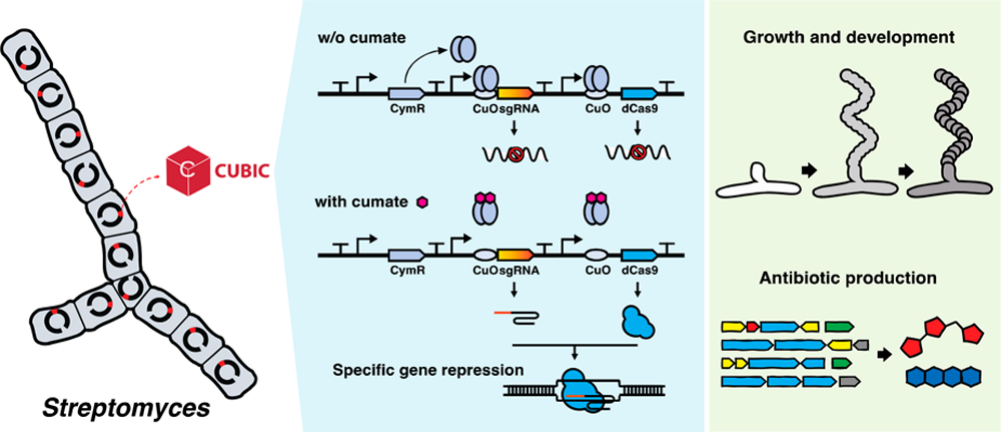

*Streptomyces*, a genus of Gram-positive bacteria, is known as nature’s medicine maker, producing a plethora of natural products that have huge benefits for human health, agriculture, and biotechnology. To take full advantage of this treasure trove of bioactive molecules, better genetic tools are required for the genetic engineering and synthetic biology of *Streptomyces*. We therefore developed CUBIC, a novel CUmate-Based Inducible CRISPR interference (CRISPRi) system that allows highly efficient and inducible gene knockdown in *Streptomyces*. Its broad application is shown by the specific and nondisruptive knockdown of genes involved in growth, development and antibiotic production in various *Streptomyces* species. To facilitate hyper-efficient plasmid construction, we adapted the Golden Gate assembly to achieve 100% cloning efficiency of the protospacers. We expect that the versatile plug-and-play CUBIC system will create new opportunities for research and innovation in the field of *Streptomyces*.

## Introduction

Streptomycetes are the most prolific source of natural bioactive substances for pharmaceutical and agrochemical applications, and producers of a wide range of industrial enzymes. These bacteria produce over half of all clinically used antibiotics, as well as a wide range of other medicinal drugs, including immunosuppressants and anticancer, antifungal, and anthelmintic drugs.^1^ To optimally harness their biosynthetic potential, we need efficient genetic manipulation and genome editing tools. However, in contrast to the well-studied unicellular microorganisms, such as *Bacillus subtilis*, *Escherichia coli*, and *Saccharomyces cerevisiae*, there are limited genetic tools available for *Streptomyces*.

Over the past decade, clustered regularly interspaced short palindromic repeats (CRISPR)/CRISPR-associated protein (Cas) systems have emerged as a powerful tool for genome editing and have revolutionized almost every aspect of biology. In *Streptomyces*, Cas9 nuclease allows the introduction of double-stranded breaks (DSBs) in their chromosome at specific locations, which can then be repaired through NHEJ pathway or homologous recombination to generate desired mutants.^2,3^ Despite the ease and efficient use, one of the foremost challenges of the CRISPR/Cas9 system is that the DSBs generated by the Cas9 nuclease may lead to chromosomal rearrangements, genomic instability, and even cell death.^4^ An alternative technology for gene regulation is CRISPR interference or CRISPRi. The catalytically dead Cas9 (dCas9) in combination with a single guide RNA (sgRNA) generates a DNA recognition complex that can interfere with binding of the RNA polymerase and transcription factors, which leads to a block in the transcription of specific genes.^5^ In *Streptomyces*, the dCas9-mediated CRISPR interference (CRISPRi) and CRISPR activation (CRISPRa) systems have been established in *S. venezuelae* for activation of biosynthetic gene clusters (BGCs).^6^ These systems had been validated for one specific *Streptomyces* species, and both lack an inducible promoter, so that interference with gene expression is permanent and not reversible. The thiostrepton-inducible promoter is the most used inducible promoter system in *Streptomyces*,^7^ but has the major disadvantage that thiostrepton induces a stress response, whereby many genes are inadvertently switched on.^8^ Similar issues exist with other native *Streptomyces* promoter systems such as those induced by tetracycline,^9^ γ-butyrolactones (GBL),^10^ and cellobiose,^11^ whereby the latter two were applied as part of CRISPRi systems. Therefore, new systems for inducible CRISPRi in *Streptomyces* are needed.

Here, we have employed an exogenous cumate-inducible gene expression system from *Pseudomonas putida*.^12,13^ The inducer cumate (4-isopropylbenzoic acid) is nontoxic to the host, highly orthogonal to *Streptomyces* metabolic pathways and inexpensive. We present the CUmate-Based Inducible CRISPRi (CUBIC) system for target-specific gene regulation in multiple *Streptomyces* species, which is a powerful and versatile new tool for the genetic engineering and study of *Streptomyces* bacteria. Furthermore, we developed a fail-proof Golden Gate method for protospacer sequences cloning, showcasing its potential for efficient generation of CRISPRi libraries through high-throughput methods.

## Results and Discussion

### Establishment of the CUBIC System in *Streptomyces*

To facilitate multifaceted genetic constructs in *Streptomyces*, we developed a hierarchical modular cloning (HMC) system (Figure S1) based on the Golden Gate cloning method^14^ that has been widely applied in the plant and microorganism synthetic biology communities.^15^ The first level of our HMC system was generated by cloning the individual DNA elements such as promoter, CDS, and terminator, flanked by *SapI* restriction sites, into plasmid pKan (Level 1). These DNA parts were then combined into a single transcriptional unit (TU), flanked by *BsaI* restriction sites, and introduced into plasmid pAmp (Level 2). A multigene assembly was then constructed by assembling the multiple TUs into site-specific integrating vectors pTHS or pPAP (Level 3)^16^ to facilitate conjugation into *Streptomyces*. Due to the lack of terminators with distinctive sequences and to avoid repetitive usage in multigene constructs, we recharacterized ten synthetic terminators published previously.^17^ Terminators L3S1P13, L3S1P47, and L3S2P21 exhibited the best performance and were employed further for multigene constructs (Figure S2). The chromatic red fluorescent protein (RFP) was used for the rapid detection of correct recombinant *E. coli* strains. In order to develop a high-performance inducible regulatory system for our CUBIC system and to showcase the HMC system, we designed four induction modules whereby the transcription of the genes for either the CymR repressor or superfolder green florescent protein (sfGFP) were controlled by either the weaker synthetic promoter SP11 or the strong one SP30.^18^ Expression of sfGFP expression in *S. venezuelae* ATCC15439 was quantified using flow cytometry. The best performance was achieved when transcription of the genes for CymR and sfGFP was driven by SP11 and SP30, respectively (Figure S3). Overall, the HMC system provides an efficient and robust multigene construction strategy, which greatly facilitates building, fine-tuning, and debugging steps in the synthetic biology approach in *Streptomyces*, particularly when considering the long design-build-test-learn (DBTL) cycle in this genus.

**Figure 1:**
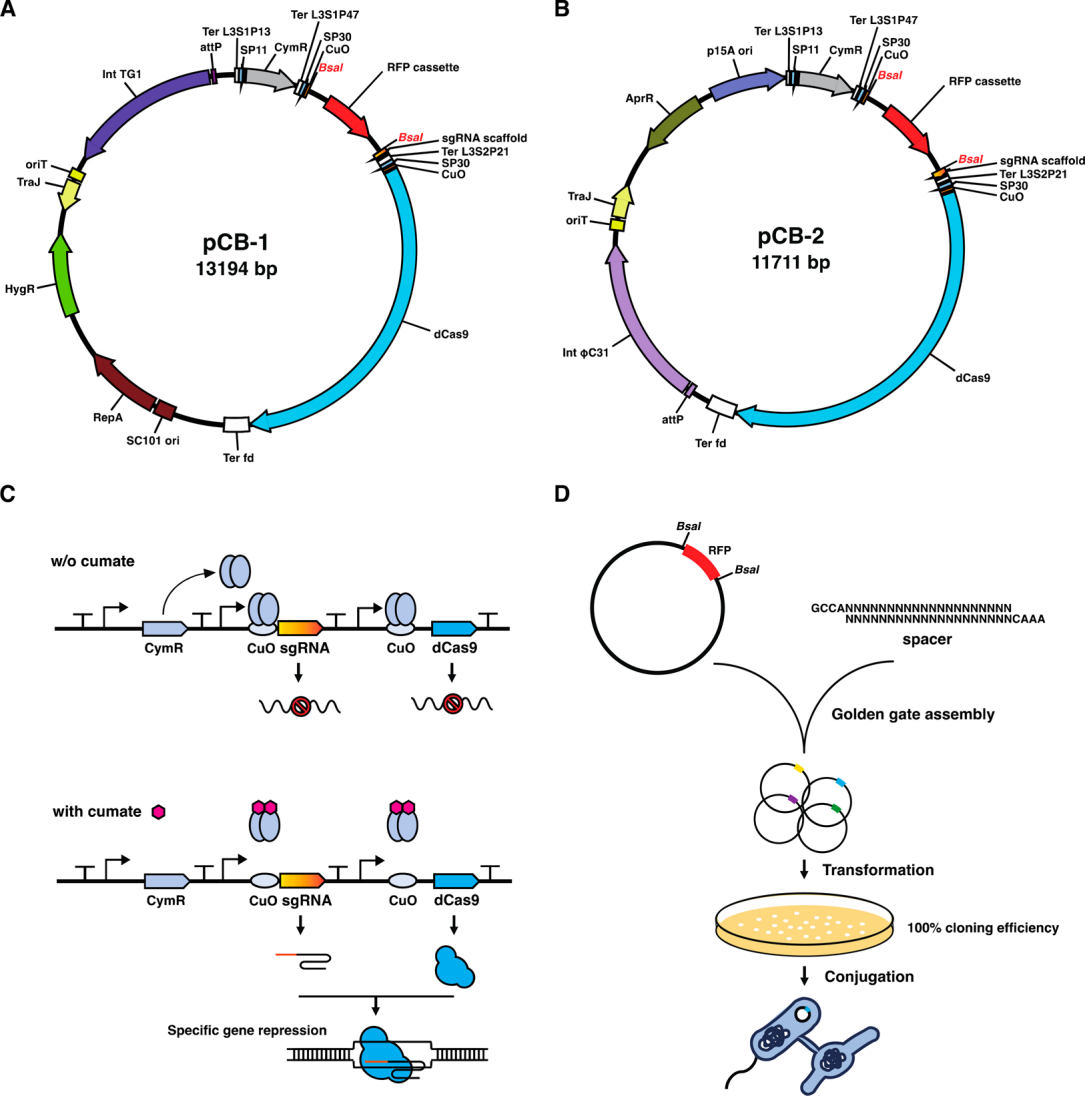
Schematic illustration of CUBIC system in *Streptomyces*. Two CUBIC plasmids pCB-1 (A) and pCB-2 (B) differ in the site-specific chromosomal integration system (TG1 and φC31 integrase-based, respectively), selection marker (conferring hygromycin and apramycin resistance, respectively), and origin of replication in *E. coli* (pSC101 and p15A, respectively). (C) In the absence of cumate, expression of sgRNA and dCas9 are tightly repressed by the binding of the repressor CymR to the cumate operator CuO located upstream of the genes for both sgRNA and dCas9. CymR repression is relieved upon the addition of cumate. The dCas9 will then be guided by the sgRNA to the target gene and switch off its transcription. (D) The workflow for construction of inducible CRISPRi mutants. In brief, the CUBIC plasmid with protospacer was generated by Golden Gate assembly and further transformed into *E. coli* ET12567 strain with 100% efficiency. Then the construct was delivered to *Streptomyces* strains via conjugation, allowing for inducible repression of target genes.

Next, we sought to establish and fine-tune the CUBIC system. For this, we first targeted *actII*-ORF4 (SCO5085), encoding the pathway-specific activator for actinorhodin (Act) biosynthesis in the model strain *S. coelicolor* M145. The effect can be readily visualized as Act is a blue-pigmented secreted antibiotic. A sgRNA targeting *actII-*ORF4 in the *act* gene cluster was selected by Geneious Prime software (Figure S4). Subsequently, it was placed downstream of the SP30-CuO promoter-operator sequence, and transcription of the gene for dCas9 was driven by different regulatory elements (SP11 or SP30 with or without CuO operator). When transcription of the gene for dCas9 was under the control of the SP30 promoter combined with the CuO operator, Act production was totally abolished in the presence of only 10 μM cumate, while wild-type production of Act was obtained in the absence of cumate (Figure S5). Conversely, Act production was still produced upon induction when the weak SP11 promoter was employed. The absence of the CuO operator in front of dCas9 also led to leaky expression (Figure S5). Based on these data, we selected two CUBIC plasmid systems (pCB-1 and pCB-2) that were based on the pTHS and pPAP backbone, respectively (Figure 1A,B). In both CUBIC plasmids, the RFP cassette flanking by two *BsaI* restriction sites in front of CRISPR RNA scaffold facilitates a plug-and-play and cost-effective strategy for spacer cloning (Figure 1D and Table S5). It is important to highlight that in this way we obtained 100% efficiency in cloning protospacers, enabling further high-throughput applications such as construction of the genome-scale CRISPRi library (Figure S6).

### Validation of CUBIC in *Streptomyces*

**Figure 2:**
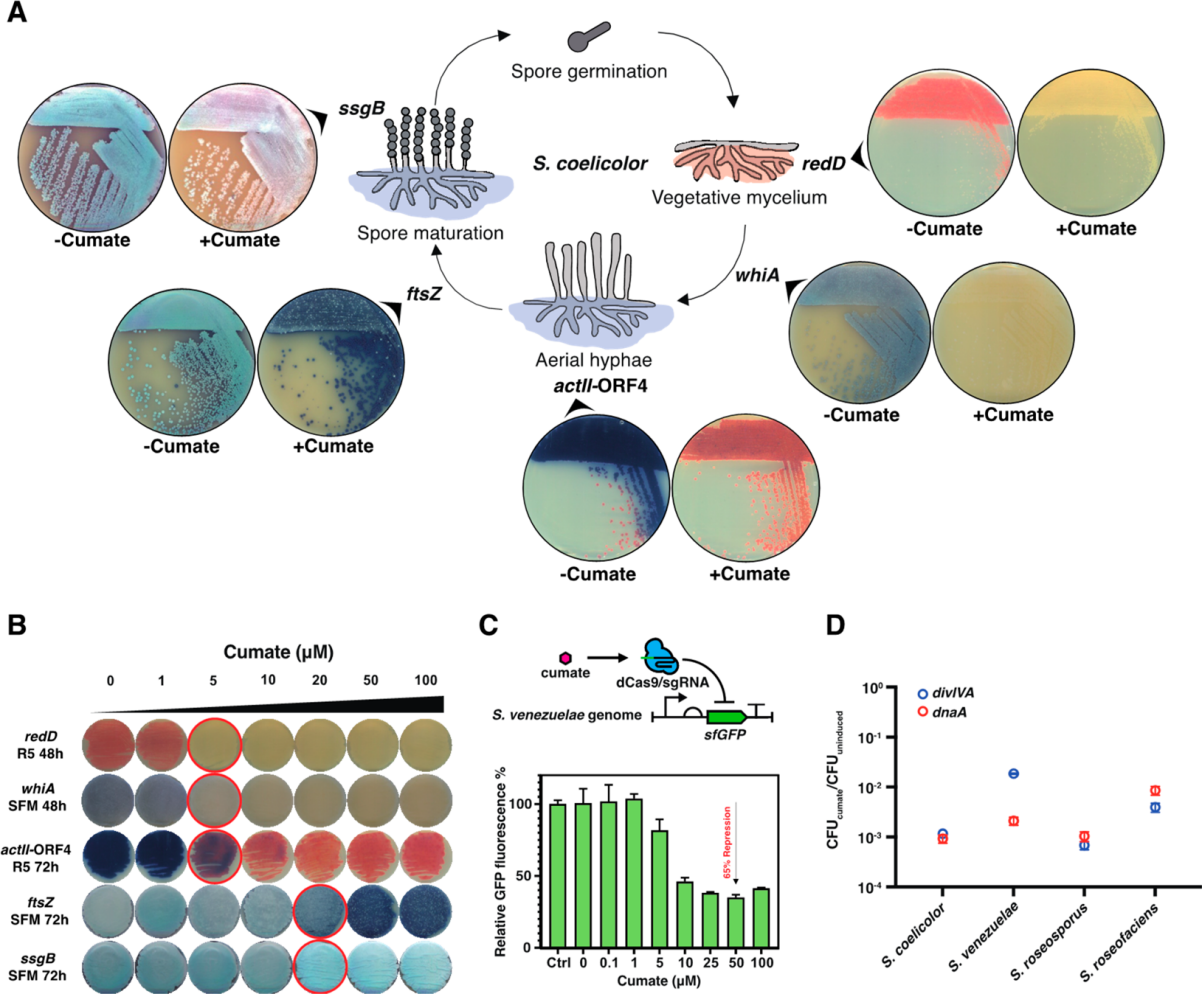
Validation of CUBIC system in various *Streptomyces* species. (A) Genes responsible for morphological development (*whiA*, *ftsZ*, and *ssgB*) or regulation of antibiotic production (*actII-*ORF4 and *redD*) in *S. coelicolor* were targeted to demonstrate the efficiency of CUBIC. *S. coelicolor* M145 strains harboring CUBIC plasmids targeting on candidate genes were cultivated on R5 or SFM agar plates with or without 100 μM cumate. (B) Concentration-dependent phenotypic changes of *S. coelicolor* M145 strains harboring CUBIC plasmids with different sgRNAs. The red circles highlight the cumate concentration for noteworthy phenotypic changes. (C) A constitutively expressed sfGFP-based reporter system integrated into the genome of *S. venezuelae* ATCC15439 was applied to quantitatively evaluate the CUBIC system. Flow cytometry of protoplasts in which the CUBIC system was induced by increasing concentrations of cumate, the control (Ctrl) showing the sfGFP fluorescence of the cell without CUBIC system; strong repression of transcription was achieved at 10 μM cumate. (D) Inducible knockdown of two genes essential for growth (*divIVA* and *dnaA*) in multiple *Streptomyces* species by CUBIC system. Spores of *Streptomyces* harboring CUBIC plasmids were spread on SFM agar plates with or without 100 μM cumate, the *y*-axis represents the value of the CFU of cumate-induced cells divided by the CFU of uninduced ones. Error bars, ±1 SD.

To further validate the CUBIC in *Streptomyces*, the system was applied to knock down the expression of genes responsible for regulation of antibiotic production and morphological differentiation in *S. coelicolor* M145 (Figure 2A). First, we applied CUBIC to silence *redD* (SCO5877), the pathway-specific activator gene for the biosynthesis of the red-pigmented prodiginines (Red). Indeed, like seen for Act when *actII*-ORF4 was targeted, no Red was produced in the presence of cumate, while Red production was normal in the absence of inducer. We also examined CUBIC for genes that play pivotal roles in *Streptomyces* life cycle, namely the cell division and morphology-related genes *ftsZ*, *ssgB*, and *whiA*. FtsZ (SCO2082) is a key protein in cell division, forming the contractile ring that recruits the cell division machinery.^19,20^ Streptomycetes have two types of cell division, namely cross-wall formation during early (vegetative) growth and more canonical cell division during developmental growth in the aerial hyphae, whereby many cell division events divide the hyphae into chains of spores.^21^ Uniquely, *ftsZ* mutants can be created in *Streptomyces*, which are devoid of septa and form sick colonies that form sparse aerial hyphae and overproduce Act.^22^ Importantly, *S. coelicolor* M145 harboring pCB1-*ftsZ*_SC_ had a phenotype very similar to that of *ftsZ* null mutants (Figure S7) after induction with cumate, while colonies looked normal without cumate. Next we applied CUBIC to interfere with the expression of *ssgB* (SCO1541), for the cell division regulator SsgB that positively controls the recruitment of FtsZ to initiate cell division during aerial growth; as a consequence, *ssgB* null mutants have a nonsporulating phenotype but produce normal aerial hyphae.^23^ Upon induction, *S. coelicolor* M145 harboring pCB1-*ssgB*_SC_ indeed produced aerial hyphae but failed to sporulate on SFM agar plates (Figure 2A), again very similar to the phenotype of the deletion mutant.^24^ Finally, we targeted *whiA* (SCO1950), encoding a master regulator for aerial growth, cell division, and chromosome segregation.^25^ Development of *S. coelicolor* M145 harboring pCB1-*whiA*_SC_ was blocked at a stage of aerial development when cumate was added, but not without (Figure 2A). Notably, the threshold cumate concentration for effective knockdown of these genes is very similar, namely a concentration between 5 μM to 20 μM (Figure 2B), which demonstrates the robustness of the CUBIC system. We then quantitatively validated the CUBIC system in *S. venezuelae* ATCC15439 harboring a constitutively expressed sfGFP by a flow cytometry-based approach.^18^ According to our data, the CUBIC system has no basal repression and is highly titratable upon adding the specified amount of cumate, obtaining 65% repression at saturating inducer concentrations (Figure 2C).

The inducible CRISPRi technology has been leveraged to investigate essential genes in diverse organisms, but not yet in *Streptomyces*.^26−28^ We therefore evaluated the feasibility of CUBIC to knockdown essential genes in multiple *Streptomyces* strains. These included two model strains *S. coelicolor* and *S. venezuelae*, the daptomycin producer *S. roseosporus* and an isolated strain *S. roseofaciens* which is used extensively in our laboratory.^29^ We first investigated *divIVA* that is essential for hyphal tip growth and is highly conserved among actinomycetes.^30^ The *divIVA* gene (SCO2077) is located in the division and cell wall (*dcw*) gene cluster containing *ftsZ* and other cell wall biosynthesis genes. Unlike unicellular bacteria like *B. subtilis*, *divIVA* cannot be deleted in multicellular filamentous *Streptomyces*. Hence, we introduced pCB1-*divIVA* plasmids, designed to target the *divIVA* gene, into various *Streptomyces* strains. Subsequently, the viability of *Streptomyces* strains carrying the corresponding pCB1-*divIVA* plasmids were determined by counting colony-forming units (CFU) on SFM agar plates in the absence and presence of 100 μM cumate, respectively. Upon induction, all the four *Streptomyces* harboring CUBIC plasmid resulted in a viable fraction of 10^–2^ to 10^–3^ (Figure 2D). Next, we applied CUBIC system to knockdown *dnaA* (SCO3879), encoding DnaA that is essential for the initiation of chromosomal replication. When CUBIC was applied to target *dnaA*, a 2-log to 3-log reduction in viability (counted as CFU) was achieved upon induction in all the four *Streptomyces* species we tested (Figure 2D). This again shows the applicability of CUBIC for efficiently silencing any gene of interest in *Streptomyces*, regardless of its indispensability. Furthermore, the CUBIC system offers a substantial benefit in preserving *Streptomyces* mutants exhibiting growth (e.g., *divIVA* and *dnaA*) and sporulation (e.g., *ssgB* and *ftsZ*) defects.

## Conclusion

In summary, a novel inducible CRISPRi system in streptomycetes designated CUBIC has been developed. The exogenous cumate-based inducible regulatory system is orthogonal in *Streptomyces* species displaying high-performance and versatility. Applications of CUBIC include analysis of gene function, modulation of natural product biosynthetic pathways, and more. We expect that CUBIC will significantly facilitate the fundamental research and drug discovery and development in the field of streptomycetes.

## Supporting Information Available

Materials and methods; scheme of HMC system (Figure S1), characterization of synthetic terminators (Figure S2), optimization of inducible system (Figure S3), design of protospacer (Figure S4), optimization of CUBIC system (Figure S5), highly efficient construction of CUBIC plasmids (Figure S6), phenotypes of representative knockout mutants (Figure S7); lists of strains, plasmids, sequences of genetic parts and primers used in this study (Tables S1–S4), cost summary of constructing CUBIC plasmids (Table S5) (PDF)

## Abbreviations

CRISPR: clustered regularly interspaced short palindromic repeats
sgRNA: single guide RNA
HMC: hierarchical modular cloning.
