# Supplementary material for: CUBIC: A Versatile Cumate-Based Inducible CRISPRi System in Streptomyces
Source: ACS Synth Biol. 2023 Oct 6;12(10):3143–7. doi: 10.1021/acssynbio.3c00464 (PMC10594651; doi:10.1021/acssynbio.3c00464)
Supplement: Supplementary file 1 — sb3c00464_si_001.pdf [file sb3c00464_si_001.pdf]

Supporting Information for

CUBIC: A Versatile CUmate-Based Inducible CRISPRi System in *Streptomyces*

Chaoxian Bai and Gilles P. van Wezel*

Institute of Biology, Leiden University, Sylviusweg 72, 2333 BE, Leiden, Netherlands.

*Corresponding author: Prof. Gilles P. van Wezel. Email: g.wezel@biology.leidenuniv.nl

Materials and Methods

Strains, Media, and Growth Conditions

The strains used in this study are listed in Table S1. *E. coli* strains were cultivated in Luria-Bertani medium at 37 °C. Soya Flour Mannitol (SFM) medium was used for sporulation and conjugation. R5 medium were used for the studies of antibiotic production in *Streptomyces*.[1] The medium was supplemented with appropriate antibiotics if necessary (kanamycin at 50 μg/ml, ampicillin at 100 μg/ml, hygromycin at 100 μg/ml, chloramphenicol at 25 μg/ml, apramycin at 50 μg/ml, and nalidixic acid at 20 μg/ml). Intergeneric transfer of plasmids from *E. coli* to *Streptomyces* strains was carried out by triparental conjugation (ET12567/pUB307 × ET12567/*oriT* plasmid × *Streptomyces*) as previously described.[1]

Hierarchical Assembly via Golden Gate Cloning in *Streptomyces*

Before assembly into complex constructs, the type IIS restriction sites (*BsaI* and *SapI*) have to be removed from DNA parts by point mutation or gene synthesis. The DNA parts (promoter, terminator, coding sequence, etc.) were firstly subcloned into Level 1 vector pKan flanking by two *SapI* restriction sites using NEBuilder®HiFi DNA Assembly Master Mix (New England BioLabs, E2621L). Level 2 plasmids were obtained via Golden Gate assembly reaction (10 μl) consisted of 20 fmol of each DNA parts, 10 fmol backbone (pAmp), 1 μl 10 × T4 DNA ligase buffer, 5 units *SapI* (New England BioLabs, R0569L), and 200 units T4 DNA Ligase (New England BioLabs, R0202L). Assembly reactions were incubated in a thermal cycler with 30 cycles consisting 5 min at 37 °C and 5 min at 16 °C, followed by a 5 min final incubation step at 60 °C and then a final 4 °C hold prior to transformation. Level 3 Plasmids were obtained via

Golden Gate assembly reaction (10 µl) consisted of 10 fmol of each Level 2 plasmids, 10 fmol backbone, 1 µl 10 × T4 DNA ligase buffer, 10 units *BsaI* -HF®v2 (New England BioLabs, R3733L), and 200 units T4 DNA Ligase (New England BioLabs, R0202L). Assembly reactions were incubated in a thermal cycler with 10 cycles consisting 5 min at 37 °C and 10 min at 16 °C, followed by a 15 min incubation step at 37 °C and 50 °C for 5 min and 80 °C for 5 min prior to transformation into *E. coli* DH5α or ET12567.

Quantification of Fluorescent Protein Expression in *Streptomyces* by Flow Cytometry

To precisely measure the expression level of fluorescent protein (sfGFP and mCherry) at single cell level in *Streptomyces*, the flow cytometry-based method was applied.[2] In the case of *S. venezuelae* ATCC15439, 50 µl overnight culture was inoculated into 1 ml YEME-TSBS (1:1) liquid medium supplemented with 2.5 mM $MgCl_2$ and 0.25% glycine in 24-well plates and shaking at 200 rpm at 30 °C for 16 hours. Protoplasts were prepared as described previously.[1] Before flow cytometry, 20 µl filtered protoplast suspension was mixed with 180 µl PBS buffer (pH 7.4) supplemented with 0.5 M NaCl, 1 mg/ml kanamycin and 5 µg/ml propidium iodide (PI). The protoplasts were then analyzed by the S3e™ Cell Sorter (Bio-Rad). The parameters of flow cytometer were set as follows: FSC-283, SSC-236, FL1-750, FL3-762 and threshold is FSC-0.10. Data was further processed by FlowJo software (version 10.4, BD).

Characterization of Synthetic Terminators in *Streptomyces*

Since only a handful of terminators (e.g., *fd* terminator) are used in *Streptomyces*, more reliable transcriptional terminators that have sufficient sequence diversity are needed for constructing

complex genetic system. Ten synthetic terminators characterized in *E. coli* were chosen to test their efficiency in *Streptomyces* (Table S3).[3] Terminator efficiency (*Te*) is calculated by:

$$Te = 1 - \left(\frac{GFP_0}{mCherry_0}\right)\left(\frac{GFP_{Term}}{mCherry_{Term}}\right)^{-1}$$

where *Term* refers to the measurements of fluorescence when the terminator is present and *0* refers to the measurement of the control in *S. venezuelae* ATCC15439 protoplasts.

CRISPR Protospacer Design

To find the "$N_{(20)}$NGG" CRISPR sites for gene repression in *Streptomyces*, the Geneious Prime software (version 2023.0.1; Biomatters Ltd.) was used according to the tutorial. Briefly, the Find CRISPR sites tool in Geneious Prime will search for all potential CRISPR sites in the selected region, and search for off-target binding sites in any given genome of interest. Only the protospacer with high specificity score (>95%) on non-template (NT) strand will be chosen. An example was given for protospacer design for SCO5085 in *S. coelicolor* M145 using Geneious Prime software.

CUBIC Plasmid Assembly

All CUBIC plasmids were constructed using a modified Golden Gate assembly method.[4] Firstly, two 24 nt oligonucleotides (final concentration 1 µM) were annealed at 95 °C for 5 min and cooled down to 4 °C at 0.1 °C/s. Secondly, the double-stranded oligonucleotide was diluted to 100 nM for phosphorylation in 10 µl reaction volume containing 3 µl diluted oligonucleotide, 1 µl 10 × T4 DNA ligase buffer and one unit T4 Polynucleotide Kinase (New England BioLabs, M0201L) and incubated at 37 °C for 1 hour. Thirdly, set up 10 µl reaction solution containing 10

fmol backbone (pCB-1 or pCB-2), 1.3 µl phosphorylated oligonucleotide, 1 µl 10 × T4 DNA ligase buffer, 2 units *BsaI* -HF®v2 (New England BioLabs, R3733L) and 40 units T4 DNA Ligase (New England BioLabs, R0202L). The Golden Gate reaction was performed in a thermal cycler for 10 cycles of 5 min at 37 °C and 10 min at 16 °C, then 15 min at 37 °C, followed by 50 °C for 5 min and 80 °C for 5 min to inactivated enzyme. Finally, the reaction mix was treated with 5 units T5 Exonuclease (New England BioLabs, M0663L) at 37 °C for 1 hour to remove incomplete ligation products. The reaction mix can be further transformed into *E. coli* DH5α or ET12567 competent cells.

Supplementary Figures

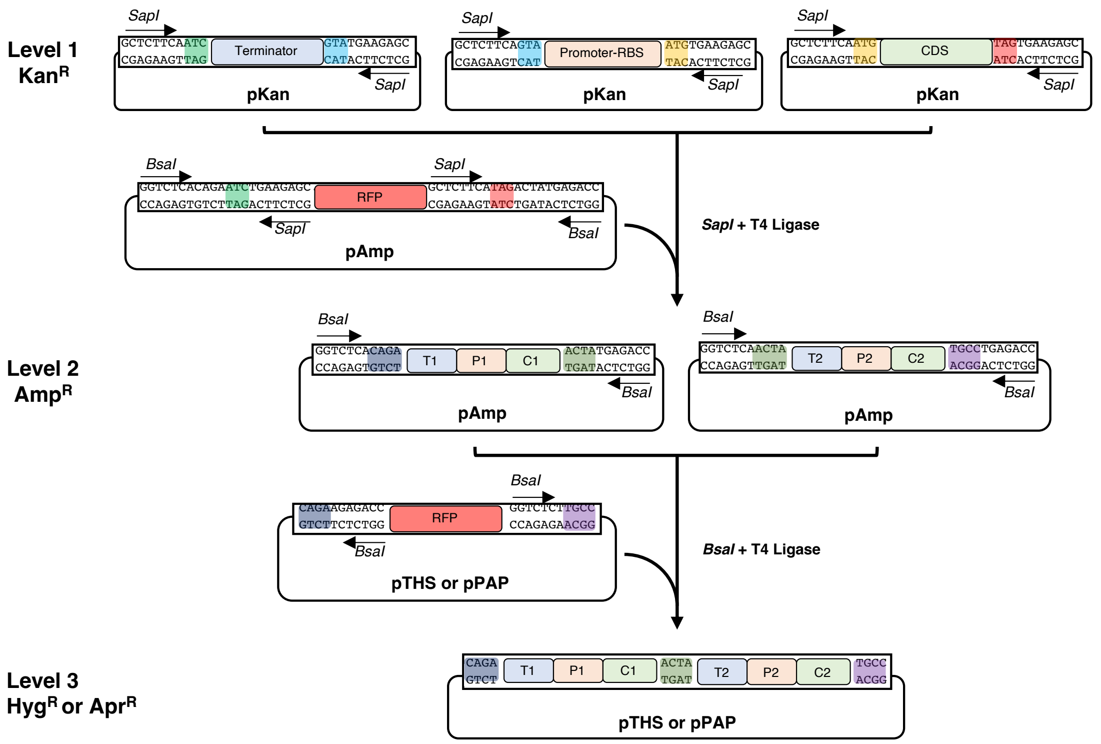

Figure S1. Schematic depiction of Hierarchical Modular Cloning (HMC) system in *Streptomyces*.

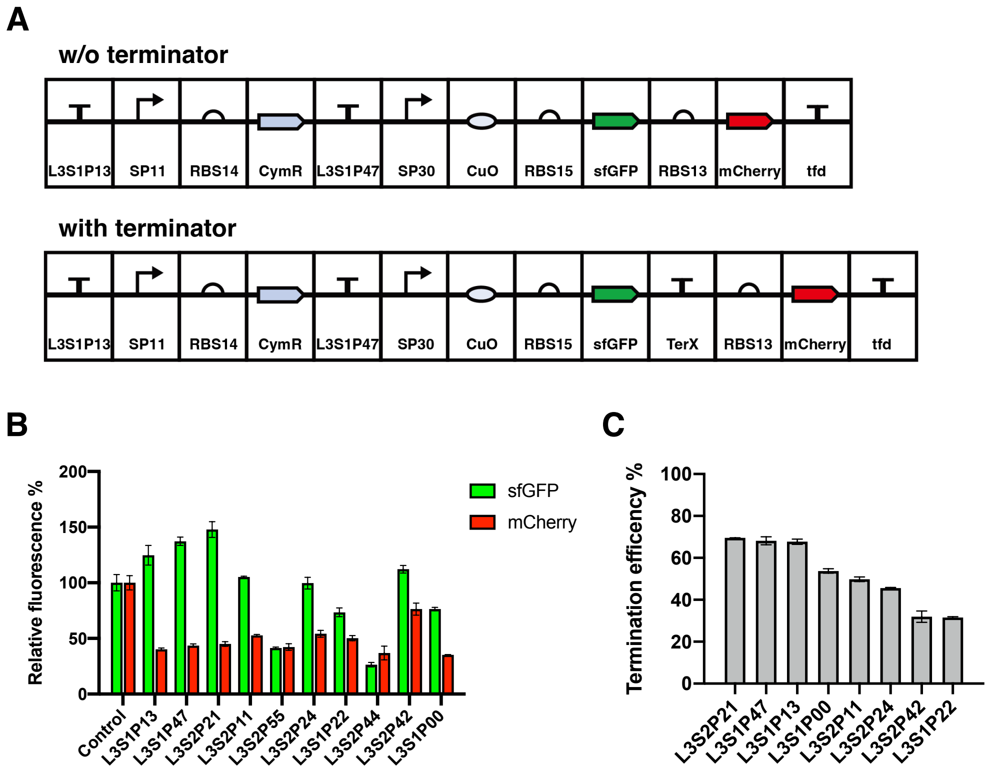

Figure S2. Characterization of synthetic terminators in *Streptomyces venezuelae* ATCC15439 by quantification of fluorescent protein expression.

(A) Ten synthetic terminators (TerX) were inserted between two fluorescent proteins (sfGFP and mCherry), while there is no terminator between fluorescent proteins in control plasmid.

(B) Measurement of sfGFP and mCherry fluorescence in *S. venezuelae* ATCC15439 protoplasts under induced state (100 μM cumate). Error bars, ± 1 SD.

(C) Termination efficiency (*Te*) of 8 synthetic terminators. Two terminators (L3S2P55 and L3S2P44) were discarded.

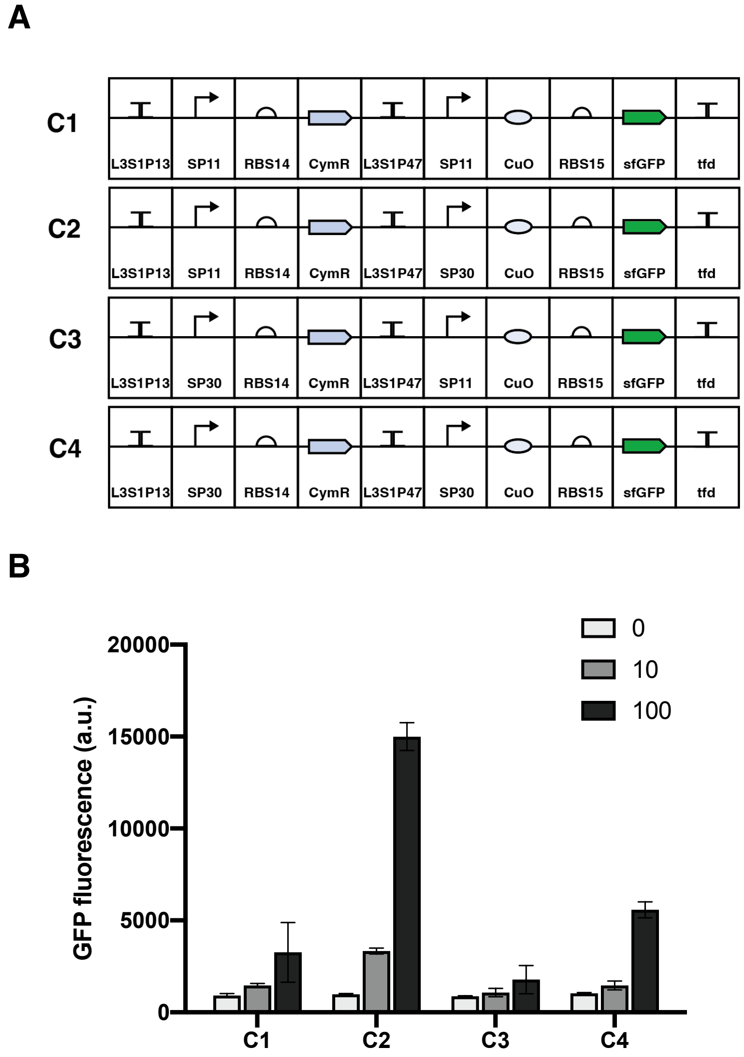

Figure S3. Optimization of cumate-based inducible system in *Streptomyces venezuelae* ATCC15439.

(A) Design of the cumate-based induction modules. Four different induction modules were designed by combining two synthetic promoters (SP11 and SP30)[2] and coding sequences (CymR and sfGFP) in pTHS plasmid.[5]

(B) The performance of induction modules was characterized by measuring sfGFP fluorescence in *S. venezuelae* ATCC15439 protoplasts. The cumate concentrations used were 0 µM, 10 µM, and 100 µM. a.u., arbitrary units. Error bars, ± 1 SD.

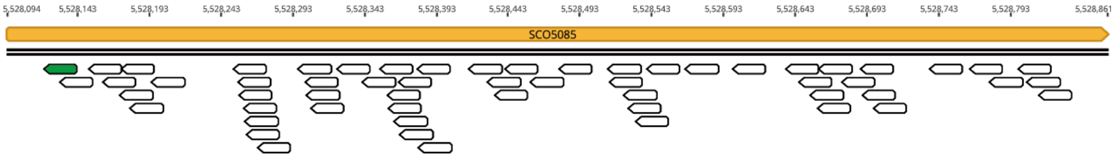

Figure S4. Example of CRISPR protospacer design using Geneious Prime.

All potential CRISPR sites on non-template (NT) strand for ActII-ORF4 (SCO5085) in *S. coelicolor* M145 are shown. The protospacer (green colored) with high specificity score (low risk of off-target effects) is selected for further CRISPRi experiment.

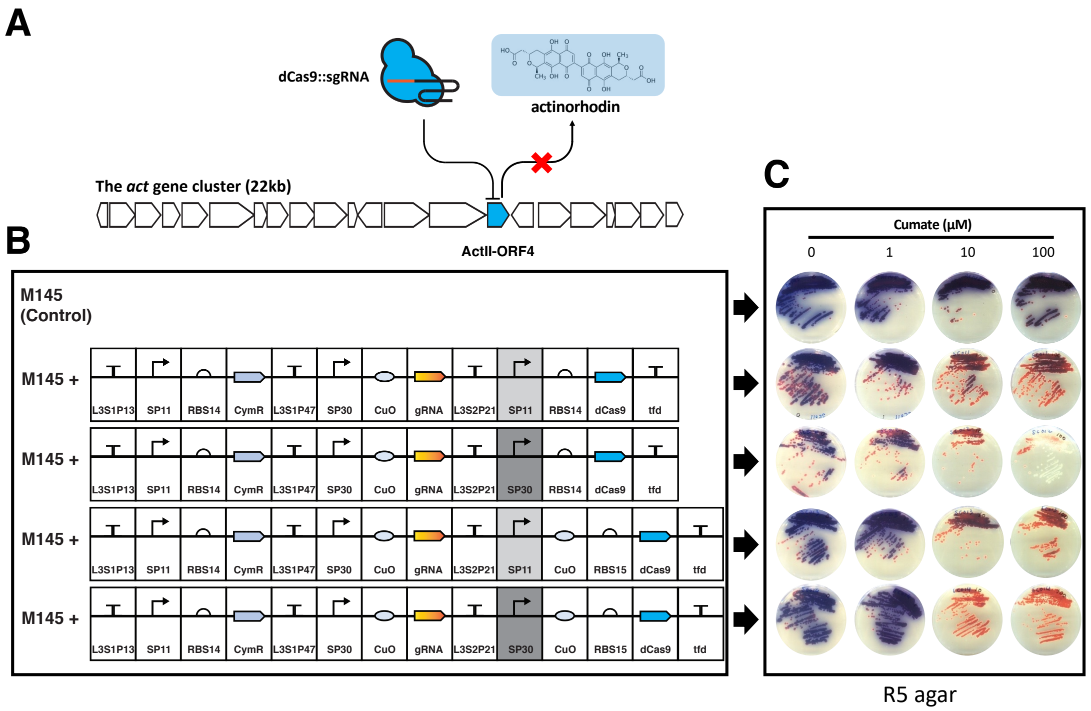

Figure S5. Optimization of CUBIC system in *S. coelicolor* M145.

(A) The pathway-specific activator ActII-ORF4 was chosen as CUBIC target to evaluate the performance of different CUBIC systems.

(B) Design of four CUBIC systems differ in the control elements of dCas9 (promoter strength in combination with or without CuO operator).

(C) Act production by *S. coelicolor* M145 harboring CUBIC plasmids was affected upon cumate induction. The combination of strong promoter SP30 and CuO operator in front of dCas9 gave the lowest leakiness and best performance.

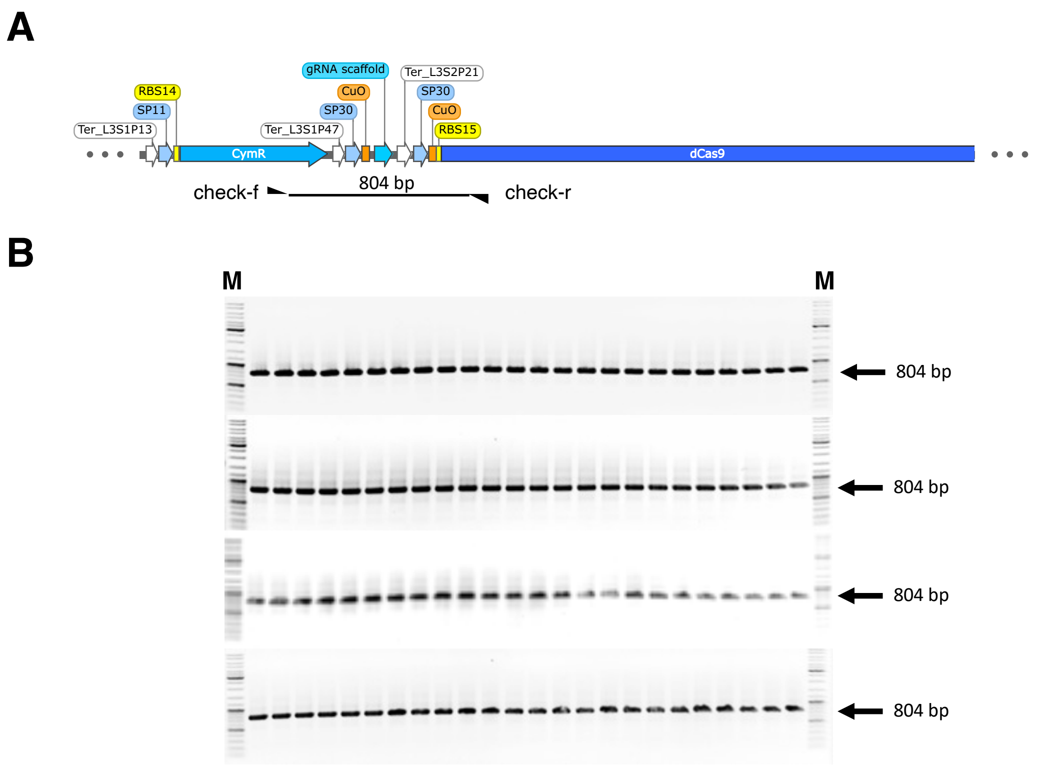

Figure S6. High-efficiency cloning of CUBIC plasmids.

(A) Schematic representation of primer set for validating positive clones with the assembled CUBIC plasmids.

(B) PCR screening results of 96 randomly picked colonies approached 100% correct clones, the marker is GeneRuler DNA Ladder Mix (Thermo Scientific, SM0334).

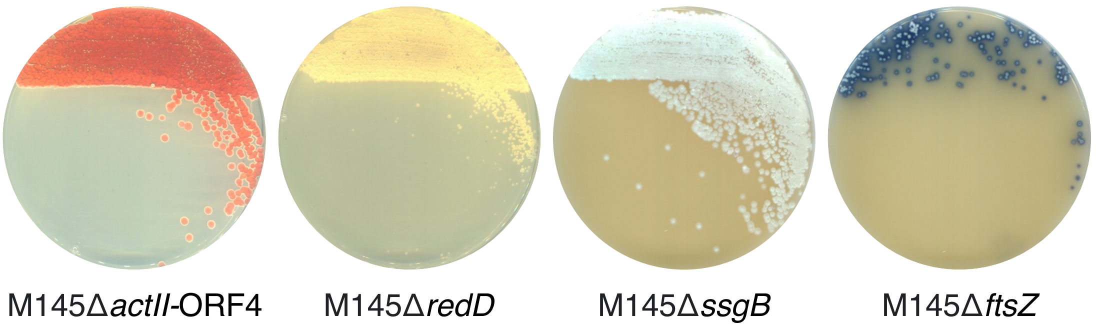

Figure S7. Phenotypes of respective mutants (*actII*-ORF4, *redD*, *ssgB* and *ftsZ*) of *S. coelicolor* M145.

Supplementary Tables

Table S1. List of strains used in this study.

Strains	Description	Source
E. coli DH5α	For routine cloning and plasmid propagation	Maintained in lab
E. coli ET12567	Methylation-deficient host	Maintained in lab
E. coli ET12567/pUB307	ET12567 carrying the self-transmissible plasmid pUB307 for triparental mating	Maintained in lab
S. coelicolor M145	Model *Streptomyces*	Maintained in lab
S. coelicolor M510	*S. coelicolor redD* mutant	Maintained in lab [6]
S. coelicolor M511	*S. coelicolor actII*-ORF4 mutant	Maintained in lab [6]
S. coelicolor Δ*ssgB*	*S. coelicolor ssgB* mutant	Maintained in lab [7]
S. coelicolor Δ*ftsZ*	*S. coelicolor ftsZ* mutant	Maintained in lab [7]
S. venezuelae ATCC15439	Model *Streptomyces*	Maintained in lab
S. venezuelae ATCC15439 (sfGFP)	Constitutively expressed sfGFP	This study
S. roseosporus ATCC31568	Daptomycin producer	Maintained in lab
S. roseifaciens MBT76	Isolated from QinLing Mountains, China	Maintained in lab [8]

Table S2. List of plasmids used in this study.

Plasmids	Description
pCB-1	pSC101 origin, Hyg^R, TG1 integrase, *oriT-traJ*, inducible CRISPRi system with RFP cassette; Golden Gate cloning vector for CUBIC system in streptomycetes
pCB-2	p15A origin, Apr^R, ϕC31 integrase, *oriT-traJ*, inducible CRISPRi system with RFP cassette; Golden Gate cloning vector for CUBIC system in streptomycetes
pKan	pUC origin, Kan^R
pKan-CymR	CymR subcloned in pKan flanking by two *SapI* restriction sites
pKan-sfGFP	sfGFP subcloned in pKan flanking by two *SapI* restriction sites
pKan-mCherry	mCherry subcloned in pKan flanking by two *SapI* restriction sites
pKan-dCas9	dCas9 subcloned in pKan flanking by two *SapI* restriction sites
pKan-SP11-RBS14	SP11 promoter with RBS14 subcloned in pKan flanking by two *SapI* restriction sites
pKan-SP30-RBS14	SP30 promoter with RBS14 subcloned in pKan flanking by two *SapI* restriction sites
pKan-SP11-CuO-RBS15	SP11 promoter with CuO operator and RBS14 subcloned in pKan flanking by two *SapI* restriction sites
pKan-SP30-CuO-RBS15	SP30 promoter with CuO operator and RBS14 subcloned in pKan flanking by two *SapI* restriction sites
pKan-Ter1	Terminator-L3S1P13 subcloned in pKan flanking by two *SapI* restriction sites
pKan-Ter2	Terminator-L3S1P47 subcloned in pKan flanking by two *SapI* restriction sites
pKan-Ter3	Terminator-L3S2P21 subcloned in pKan flanking by two *SapI* restriction sites
pKan-Ter4	Terminator-L3S2P11 subcloned in pKan flanking by two *SapI* restriction sites
pKan-Ter5	Terminator-L3S2P55 subcloned in pKan flanking by two *SapI* restriction sites
pKan-Ter6	Terminator-L3S2P24 subcloned in pKan flanking by two *SapI* restriction sites
pKan-Ter7	Terminator-L3S1P22 subcloned in pKan flanking by two *SapI* restriction sites
pKan-Ter8	Terminator-L3S2P44 subcloned in pKan flanking by two *SapI* restriction sites
pKan-Ter9	Terminator-L3S2P42 subcloned in pKan flanking by two *SapI* restriction sites
pKan-Ter10	Terminator-L3S1P00 subcloned in pKan flanking by two *SapI* restriction sites
pKan-sgRNA$_{actII\text{-}orf4}$	SP30 promoter and CuO upstream of sgRNA$_{actII\text{-}orf4}$ in pKan flanking by two *SapI* restriction sites
pAmp	pUC origin, Amp^R
pAmp-caga-RFP-acta	pAmp with RFP flanking by two *BsaI* restriction sites
pAmp-acta-RFP-tgcc	pAmp with RFP flanking by two *BsaI* restriction sites
pAmp-tgcc-RFP-gagc	pAmp with RFP flanking by two *BsaI* restriction sites
TU001	pAmp-L3S1P13-SP11-RBS14-CymR
TU002	pAmp-L3S1P13-SP30-RBS14-CymR
TU004	pAmp-L3S1P13-SP11-CuO-RBS15-sfGFP
TU005	pAmp-L3S1P13-SP30-CuO-RBS15-sfGFP
TU023	pAmp-mCherry-Ctrl
TU024	pAmp-L3S1P13-mCherry
TU025	pAmp-L3S1P47-mCherry
TU026	pAmp-L3S2P21-mCherry
TU027	pAmp-L3S2P11-mCherry
TU028	pAmp-L3S2P55-mCherry
TU029	pAmp-L3S2P24-mCherry
TU030	pAmp-L3S1P22-mCherry
TU031	pAmp-L3S2P44-mCherry
TU032	pAmp-L3S2P42-mCherry
TU033	pAmp-L3S1P00-mCherry
TU015	pAmp-L3S1P47-SP30-CuO-sgRNA$_{actII\text{-}orf4}$
TU035	pAmp-L3S2P21-SP11-RBS14-dCas9
TU036	pAmp-L3S2P21-SP30-RBS14-dCas9
TU037	pAmp-L3S2P21-SP11-CuO-RBS15-dCas9
TU038	pAmp-L3S2P21-SP30-CuO-RBS15-dCas9
pTHS	pSC101 origin, Hyg^R, TG1 integrase, *oriT-traJ.*
pPAP	p15A origin, Apr^R, ϕC31 integrase, *oriT-traJ.*
pPAP-acta-RFP-tgcc-tfd	pPAP with RFP flanking by two *BsaI* restriction sites
pPAP-caga-RFP-gagc-tfd	pPAP with RFP flanking by two *BsaI* restriction sites
pTHS-caga-RFP-tgcc-tfd	pTHS with RFP flanking by two *BsaI* restriction sites

pTHS-caga-RFP-gagc-tfd	pTHS with RFP flanking by two *BsaI* restriction sites
pPAP-sfGFP	pPAP-SP30-RBS14-sfGFP-tfd
pTHS-sfGFP-mCherry	pTHS-SP11-CymR-SP30-CuO-sfGFP-mCherry-tfd, terminator control plasmid
pTHS-Ter1	pTHS-SP11-CymR-SP30-CuO-sfGFP-L3S1P13-mCherry-tfd
pTHS-Ter2	pTHS-SP11-CymR-SP30-CuO-sfGFP-L3S1P47-mCherry-tfd
pTHS-Ter3	pTHS-SP11-CymR-SP30-CuO-sfGFP-L3S2P21-mCherry-tfd
pTHS-Ter4	pTHS-SP11-CymR-SP30-CuO-sfGFP-L3S2P11-mCherry-tfd
pTHS-Ter5	pTHS-SP11-CymR-SP30-CuO-sfGFP-L3S2P55-mCherry-tfd
pTHS-Ter6	pTHS-SP11-CymR-SP30-CuO-sfGFP-L3S2P24-mCherry-tfd
pTHS-Ter7	pTHS-SP11-CymR-SP30-CuO-sfGFP-L3S1P22-mCherry-tfd
pTHS-Ter8	pTHS-SP11-CymR-SP30-CuO-sfGFP-L3S2P44-mCherry-tfd
pTHS-Ter9	pTHS-SP11-CymR-SP30-CuO-sfGFP-L3S2P42-mCherry-tfd
pTHS-Ter10	pTHS-SP11-CymR-SP30-CuO-sfGFP-L3S1P00-mCherry-tfd
pTHS-C1	pTHS-SP11-CymR-SP11-sfGFP-tfd
pTHS-C2	pTHS-SP11-CymR-SP30-sfGFP-tfd
pTHS-C3	pTHS-SP30-CymR-SP11-sfGFP-tfd
pTHS-C4	pTHS-SP30-CymR-SP30-sfGFP-tfd
pTHS-CRISPRi-t1	pTHS-SP11-CymR-SP30-CuO-sgRNA$_{actII\text{-}orf4}$-SP11-dCas9-tfd
pTHS-CRISPRi-t2	pTHS-SP11-CymR-SP30-CuO-sgRNA$_{actII\text{-}orf4}$-SP30-dCas9-tfd
pTHS-CRISPRi-t3	pTHS-SP11-CymR-SP30-CuO-sgRNA$_{actII\text{-}orf4}$-SP11-CuO-dCas9-tfd
pTHS-CRISPRi-t4	pTHS-SP11-CymR-SP30-CuO-sgRNA$_{actII\text{-}orf4}$-SP30-CuO-dCas9-tfd
pCB1-sfGFP	CUBIC plasmid targeted on *sfGFP*
pCB1-red*D*$_{SC}$	CUBIC plasmid targeted on *redD* in *S. coelicolor*
pCB1-ftsZ$_{SC}$	CUBIC plasmid targeted on *ftsZ* in *S. coelicolor*
pCB1-ssgB$_{SC}$	CUBIC plasmid targeted on *ssgB* in *S. coelicolor*
pCB1-whiA$_{SC}$	CUBIC plasmid targeted on *whiA* in *S. coelicolor*
pCB1-divIVA$_{SC}$	CUBIC plasmid targeted on *divIVA* in *S. coelicolor*
pCB1-divIVA$_{SV}$	CUBIC plasmid targeted on *divIVA* in *S. venezuelae*
pCB1-divIVA$_{SRS}$	CUBIC plasmid targeted on *divIVA* in *S. roseosporus*
pCB1-divIVA$_{SRF}$	CUBIC plasmid targeted on *divIVA* in *S. roseifaciens*
pCB1-dnaA$_{SC}$	CUBIC plasmid targeted on *dnaA* in *S. coelicolor*
pCB1-dnaA$_{SV}$	CUBIC plasmid targeted on *dnaA* in *S. venezuelae*
pCB1-dnaA$_{SRS}$	CUBIC plasmid targeted on *dnaA* in *S. roseosporus*
pCB1-dnaA$_{SRF}$	CUBIC plasmid targeted on *dnaA* in *S. roseifaciens*

Table S3. Sequences of regulatory elements, coding sequences and terminators.

Part	Sequence	Ref.
	**Terminator**	
**L3S1P13**	AACAATAAGGCCTCCCTAACGGGGGGCCTTTTTTATTGATAACAAAA	3
**L3S1P47**	TTTTCGAAAAAAGGCCTCCCAAATCGGGGGGCCTTTTTTTATAGCAACAAAA	
**L3S2P21**	CTCGGTACCAAATTCCAGAAAAGAGGCCTCCCGAAAGGGGGGCCTTTTTTCGTTTT<br>GGTCC	
**L3S2P11**	CTCGGTACCAAATTCCAGAAAAGAGACGCTTTCGAGCGTCTTTTTTCGTTTTGGTCC	
**L3S2P55**	CTCGGTACCAAAGACGAACAATAAGACGCTGAAAAGCGTCTTTTTTCGTTTTGGTCC	
**L3S2P24**	CTCGGTACCAAATTCCAGAAAAGACACCCGAAAGGGTGTTTTTTCGTTTTGGTCC	
**L3S1P22**	GACGAACAATAAGGCCGCAAATCGCGGCCTTTTTTATTGATAACAAAA	
**L3S2P44**	CTCGGTACCAAACCAATTATTGAAGACGCTGAAAAGCGTCTTTTTTTGTTTCGGTCC	
**L3S2P42**	CTCGGTACCAAAGAAAAATAAAAAGACGCTGAAAAGCGTCTTTTTATTTTTCGGTCC	
**L3S1P00**	GACGAACAATAAGGGGAGCGGGAAACCGCTCCCCTTTTTTATTGATAACAAAA	
**fd ter**	GATCCCGCAAAAGCGGCCTTTGACTCCCTGCAAGCCTCAGCGACCGAATATATCGG<br>TTATGCGTGGGCGATGGTTGTTGTCATTGTCGGCGCAACTATCGGTATCAAGCTGTT<br>TAAGAAATTCACCTCGAAAGCAAGCTGATAAACCGATACAATTAAAGGCTCCTTTTGG<br>AGCCTTTTTTTTTTGGAGATTTTCAACGTGAAAAAATTATTATTCGCAATTCCTTTAGTTG<br>TTCCTTTCTATTCTCACTCCGCTGAAACTGTTGAAAGTTGTTTAGCAAAACCTCATAC<br>AGAAAATTCA	9
	**Coding sequence**	
**CymR**	ATGATCATGTCCCCCAAGCGGCGGACCCAGGCGGAGCGGGCGATGGAAACCCAGG<br>GCAAGCTGATCGCGGCGGCCCTCGGCGTCCTGCGCGAGAAGGGCTACGCCGGCT<br>TCCGGATCGCGGACGTCCCGGGCGCCGCGGGCGTGTCGCGCGGCGCCCAGAGC<br>CACCACTTCCCCACCAAGCTCGAGCTGCTCCTGGCGACCTTCGAGTGGCTGTACGA<br>GCAGATCACCGAGCGCTCCCGGGCCCGCCTCGCGAAGCTGAAGCCGGAGGACGA<br>CGTCATCCAGCAGATGCTCGACGACGCCGCGGAGTTCTTCCTGGACGACGACTTCT<br>CCATCTCGCTCGACCTGATCGTCGCCGCGGACCGGGACCCCGCCCTGCGCGAGG<br>GCATCCAGCGGACCGTGGAGCGGAACCGCTTCGTCGTGGAGGACATGTGGCTCGG<br>CGTCCTGGTGAGCCGGGGCCTGTCCCGCGACGACGCCGAGGACATCCTCTGGCT<br>GATCTTCAACTCCGTCCGCGGCCTCGCCGTGCGGTCGCTGTGGCAGAAGGACAAG<br>GAGCGGTTCGAGCGCGTGCGGAACAGCACCCTGGAGATCGCCCGGGAGCGGTAC<br>GCCAAGTTCAAGCGGTGA	5
**sfGFP**	ATGCGTAAAGGCGAGGAGCTGTTCACTGGTGTCGTCCCTATTCTGGTGGAACTGGA<br>TGGTGATGTCAACGGTCATAAGTTTTCCGTGCGTGGCGAGGGTGAAGGTGACGCAA<br>CTAATGGTAAACTGACGCTGAAGTTCATCTGTACTACTGGTAAACTGCCGGTACCTTG<br>GCCGACTCTGGTAACGACGCTGACTTATGGTGTTCAGTGCTTTGCTCGTTATCCGGA<br>CCATATGAAGCAGCATGACTTCTTCAAGTCCGCCATGCCGGAAGGCTATGTGCAGGA<br>ACGCACGATTTCCTTTAAGGATGACGGCACGTACAAAACGCGTGCGGAAGTGAAATT<br>TGAAGGCGATACCCTGGTAAACCGCATTGAGCTGAAAGGCATTGACTTTAAAGAAGA<br>CGGCAATATCCTGGGCCATAAGCTGGAATACAATTTTAACAGCCACAATGTTTACATC<br>ACCGCCGATAAACAAAAAAATGGCATTAAAGCGAATTTTAAAATTCGCCACAACGTGG<br>AGGATGGCAGCGTGCAGCTGGCTGATCACTACCAGCAAAACACTCCAATCGGTGAT<br>GGTCCTGTTCTGCTGCCAGACAATCACTATCTGAGCACGCAAAGCGTTCTGTCTAAA<br>GATCCGAACGAGAAACGCGATCATATGGTTCTGCTGGAGTTCGTAACCGCAGCGGG<br>CATCACGCATGGTATGGATGAACTGTACAAATGA	2
**mCherry**	ATGGTCAGCAAGGGCGAGGAGGACAACATGGCCATCATCAAGGAGTTCATGCGCTT<br>CAAGGTGCACATGGAGGGCTCCGTGAACGGCCACGAGTTCGAGATCGAGGGCGAG<br>GGCGAGGGCCGCCCCTACGAGGGCACCCAGACCGCCAAGCTGAAGGTGACCAAG<br>GGTGGCCCCCTGCCCTTCGCCTGGGACATCCTGTCCCCTCAGTTCATGTACGGCTC<br>CAAGGCCTACGTGAAGCACCCCGCCGACATCCCCGACTACTTGAAGCTGTCCTTCC<br>CCGAGGGCTTCAAGTGGGAGCGCGTGATGAACTTCGAGGACGGCGGCGTGGTGA<br>CCGTGACCCAGGACTCCTCCCTGCAGGACGGCGAGTTCATCTACAAGGTGAAGCT<br>GCGCGGCACCAACTTCCCCTCCGACGGCCCCGTAATGCAGAAGAAGACCATGGGC<br>TGGGAGGCCTCCTCCGAGCGGATGTACCCCGAGGACGGCGCCCTGAAGGGCGAG<br>ATCAAGCAGAGGCTGAAGCTGAAGGACGGCGGCCACTACGACGCTGAGGTCAAGA<br>CCACCTACAAGGCCAAGAAGCCCGTGCAGCTGCCCGGCGCCTACAACGTCAACATC<br>AAGTTGGACATCACCTCCCACAACGAGGACTACACCATCGTGGAACAGTACGAACG<br>CGCCGAGGGCCGCCACTCCACCGGCGGCATGGACGAGCTGTACAAGTAA	

**dCas9**	ATGGACAAGAAGTACTCCATCGGCCTCGCGATCGGCACCAACTCCGTGGGCTGGG<br>CGGTCATCACCGACGAGTACAAGGTCCCCTCCAAGAAGTTCAAGGTCCTGGGCAAC<br>ACCGACCGGCACTCGATCAAGAAGAACCTGATCGGCGCCCTGCTGTTCGACAGCG<br>GCGAGACGGCCGAGGCGACCCGCCTGAAGCGGACCGCGCGTCGCCGCTACACCC<br>GGCGCAAGAACCGCATCTGCTACCTGCAGGAAATCTTCTCCAACGAGATGGCCAAG<br>GTGGACGACTCGTTCTTCCACCGCCTGGAGGAGAGCTTCCTGGTGGAGGAGGACA<br>AGAAGCACGAGCGCCACCCGATCTTCGGCAACATCGTGGACGAGGTGGCCTACCA<br>CGAGAAGTACCCCACCATCTACCACCTCCGCAAGAAGCTGGTGGACTCGACCGACA<br>AGGCGGACCTGCGGCTCATCTACCTGGCCCTCGCGCACATGATCAAGTTCCGCGG<br>CCACTTCCTCATCGAGGGCGACCTGAACCCGGACAACTCCGACGTGGACAAGCTG<br>TTCATCCAGCTGGTGCAGACCTACAACCAGCTGTTCGAGGAGAACCCCATCAACGC<br>CAGCGGCGTGGACGCCAAGGCGATCCTCTCCGCGCGCCTGAGCAAGTCCCGGCG<br>CCTGGAGAACCTCATCGCCCAGCTGCCGGGCGAGAAGAAGAACGGCCTCTTCGGC<br>AACCTGATCGCGCTGTCGCTCGGCCTGACCCCCAACTTCAAGAGCAACTTCGACCT<br>GGCCGAGGACGCGAAGCTCCAGCTGTCCAAGGACACCTACGACGACGACCTGGAC<br>AACCTGCTCGCCCAGATCGGCGACCAGTACGCGGACCTCTTCCTGGCCGCGAAGA<br>ACCTCTCGGACGCCATCCTGCTCAGCGACATCCTGCGGGTCAACACCGAGATCACC<br>AAGGCCCCGCTGTCGGCGAGCATGATCAAGCGGTACGACGAGCACCACCAGGACC<br>TGACCCTGCTCAAGGCCCTCGTGCGCCAGCAGCTGCCCGAGAAGTACAAGGAAAT<br>CTTCTTCGACCAGTCCAAGAACGGCTACGCCGGCTACATCGACGGCGGCGCGTCG<br>CAGGAGGAGTTCTACAAGTTCATCAAGCCGATCCTGGAGAAGATGGACGGCACCGA<br>GGAGCTGCTCGTCAAGCTGAACCGCGAGGACCTGCTCCGCAAGCAGCGGACCTTC<br>GACAACGGCTCCATCCCGCACCAGATCCACCTGGGCGAGCTCCACGCCATCCTCC<br>GGCGCCAGGAGGACTTCTACCCCTTCCTGAAGGACAACCGCGAGAAGATCGAGAA<br>GATCCTGACCTTCCGCATCCCGTACTACGTCGGCCCCCTGGCCCGCGGCAACTCCC<br>GGTTCGCGTGGATGACCCGGAAGTCGGAGGAGACGATCACCCCGTGGAACTTCGA<br>GGAGGTCGTGGACAAGGGCGCGTCCGCGCAGTCGTTCATCGAGCGCATGACCAAC<br>TTCGACAAGAACCTCCCGAACGAGAAGGTCCTGCCCAAGCACTCCCTGCTCTACGA<br>GTACTTCACCGTGTACAACGAGCTGACCAAGGTCAAGTACGTGACCGAGGGCATGC<br>GGAAGCCGGCCTTCCTGTCGGGCGAGCAGAAGAAGGCGATCGTGGACCTGCTGTT<br>CAAGACCAACCGCAAGGTCACCGTGAAGCAGCTGAAGGAGGACTACTTCAAGAAGA<br>TCGAGTGCTTCGACTCCGTCGAGATCAGCGGCGTGGAGGACCGCTTCAACGCCTC<br>CCTGGGCACCTACCACGACCTGCTCAAGATCATCAAGGACAAGGACTTCCTCGACA<br>ACGAGGAGAACGAGGACATCCTGGAGGACATCGTCCTCACCCTGACCCTCTTCGAG<br>GACCGCGAGATGATCGAGGAGCGGCTCAAGACCTACGCCCACCTGTTCGACGACA<br>AGGTGATGAAGCAGCTGAAGCGTCGCCGCTACACCGGCTGGGGCCGCCTCTCCCG<br>GAAGCTGATCAACGGCATCCGGGACAAGCAGAGCGGCAAGACCATCCTGGACTTC<br>CTCAAGTCCGACGGCTTCGCCAACCGCAACTTCATGCAGCTCATCCACGACGACAG<br>CCTGACCTTCAAGGAGGACATCCAGAAGGCCCAGGTGTCGGGCCAGGGCGACAGC<br>CTCCACGAGCACATCGCCAACCTGGCGGGCTCCCCGGCGATCAAGAAGGGCATCC<br>TCCAGACCGTCAAGGTCGTGGACGAGCTGGTCAAGGTGATGGGCCGCCACAAGCC<br>CGAGAACATCGTGATCGAGATGGCCCGGGAGAACCAGACCACCCAGAAGGGCCAG<br>AAGAACTCGCGCGAGCGGATGAAGCGGATCGAGGAGGGCATCAAGGAGCTCGGCA<br>GCCAGATCCTGAAGGAGCACCCGGTCGAGAACACCCAGCTGCAGAACGAGAAGCT<br>GTACCTCTACTACCTGCAGAACGGCCGCGACATGTACGTGGACCAGGAGCTCGACA<br>TCAACCGGCTGTCCGACTACGACGTGGACGCGATCGTGCCGCAGTCCTTCCTGAA<br>GGACGACTCGATCGACAACAAGGTCCTGACCCGCTCGGACAAGAACCGGGGCAAG<br>TCCGACAACGTGCCCTCGGAGGAGGTCGTGAAGAAGATGAAGAACTACTGGCGCC<br>AGCTGCTCAACGCCAAGCTCATCACCCAGCGCAAGTTCGACAACCTGACCAAGGCC<br>GAGCGGGGCGGCCTGAGCGAGCTCGACAAGGCGGGCTTCATCAAGCGCCAGCTG<br>GTCGAGACGCGGCAGATCACCAAGCACGTGGCCCAGATCCTGGACTCCCGGATGA<br>ACACCAAGTACGACGAGAACGACAAGCTGATCCGCGAGGTCAAGGTGATCACCCTC<br>AAGAGCAAGCTGGTGTCCGACTTCCGCAAGGACTTCCAGTTCTACAAGGTCCGGGA<br>GATCAACAACTACCACCACGCCCACGACGCGTACCTGAACGCCGTCGTGGGCACC<br>GCGCTGATCAAGAAGTACCCGAAGCTGGAGTCCGAGTTCGTCTACGGCGACTACAA<br>GGTCTACGACGTGCGCAAGATGATCGCCAAGAGCGAGCAGGAGATCGGCAAGGCC<br>ACCGCGAAGTACTTCTTCTACTCCAACATCATGAACTTCTTCAAGACCGAGATCACC<br>CTGGCCAACGGCGAGATCCGCAAGCGGCCCCTGATCGAGACGAACGGCGAGACG<br>GGCGAGATCGTCTGGGACAAGGGCCGCGACTTCGCCACCGTCCGGAAGGTGCTGT<br>CGATGCCGCAGGTCAACATCGTGAAGAAGACCGAGGTGCAGACCGGCGGCTTCAG<br>CAAGGAGTCCATCCTCCCCAAGCGCAACAGCGACAAGCTGATCGCCCGGAAGAAG<br>GACTGGGACCCGAAGAAGTACGGCGGCTTCGACAGCCCCACCGTCGCCTACTCCG<br>TGCTGGTCGTGGCGAAGGTCGAGAAGGGCAAGAGCAAGAAGCTGAAGTCCGTGAA<br>GGAGCTGCTCGGCATCACCATCATGGAGCGCTCCTCGTTCGAGAAGAACCCGATCG<br>ACTTCCTGGAGGCCAAGGGCTACAAGGAGGTCAAGAAGGACCTCATCATCAAGCTG<br>CCCAAGTACAGCCTGTTCGAGCTGGAGAACGGCCGCAAGCGGATGCTCGCCTCCG<br>CGGGCGAGCTGCAGAAGGGCAACGAGCTGGCCCTCCCGTCGAAGTACGTCAACTT<br>CCTGTACCTCGCGTCCCACTACGAGAAGCTGAAGGGCTCGCCCGAGGACAACGAG<br>CAGAAGCAGCTGTTCGTGGAGCAGCACAAGCACTACCTGGACGAGATCATCGAGCA	

	GATCAGCGAGTTCAGCAAGCGCGTCATCCTGGCCGACGCGAACCTCGACAAGGTG CTGTCCGCCTACAACAAGCACCGCGACAAGCCGATCCGGGAGCAGGCGGAGAACA TCATCCACCTGTTCACCCTCACCAACCTGGGCGCCCCCGCCGCGTTCAAGTACTTC GACACCACCATCGACCGCAAGCGGTACACCTCCACCAAGGAGGTCCTCGACGCGA CCCTGATCCACCAGAGCATCACCGGCCTGTACGAGACGCGCATCGACCTGTCCCAG CTCGGCGGCGACTGA	
**Regulatory element**		
**SP11**	TGTTCACATTCGAACCGTCTCTGCTTTGACAATGGCTTGAATTGGGGTGTAAAGTCG TGGCCA	2
**SP30**	TGTTCACATTCGAACCGTCTCTGCTTTGACATCGTGTGGCGCTTGGGTGTAAAGTCG TGGCCA	2
**CuO**	AACAAACAGACAATCTGGTCTGTTTGTATTAT	5
**RBS14**	CCTAACGAGGAGATCGGTTC	2
**RBS15**	TCTAAGTAAGGAGTGTCCAT	2

Table S4. Primers used in this study.

Name	Sequence (5' to 3')	Plasmid
**Genetic parts**		
pKan_L3S1P13-F	cctaacggggggcctttttttattgataacaaaagtatgaagagcgggacagtaagacgggtaag	pKan-Ter1
pKan_L3S1P13-R	aaaaaaggccccccgttagggaggccttattgttgattgaagagcacggttatccacagaatcag	
pKan_L3S1P47-F	caaatcggggggcctttttttatagcaacaaaagtatgaagagcgggacagtaagacgggtaag	pKan-Ter2
pKan_L3S1P47-R	aaaaaaggccccccgatttgggaggcctttttttcgaaaagattgaagagcacggttatccacaga atcag	
pKan_L3S2P21-F	aagaggcctcccgaaaggggggcctttttttcgttttggtccgtatgaagagcgggacag	pKan-Ter3
pKan_L3S2P21-R	ccctttcgggaggcctcttttctggaatttggtaccgaggattgaagagcacggttatc	
pKan_L3S2P11-F	agaaaagagacgctttcgagcgtcttttttcgttttggtccgtatgaagagcgggacag	pKan-Ter4
pKan_L3S2P11-R	acgctcgaaagcgtctcttttctggaatttggtaccgaggattgaagagcacggttatc	
pKan_L3S2P55-F	aacaataagacgctgaaaagcgtcttttttcgttttggtccgtatgaagagcgggacag	pKan-Ter5
pKan_L3S2P55-R	acgcttttcagcgtcttattgttcgtctttggtaccgaggattgaagagcacggttatc	
pKan_L3S2P24-F	ccagaaaagacacccgaaagggtgtttttcgttttggtccgtatgaagagcgggacag	pKan-Ter6
pKan_L3S2P24-R	acaccctttcgggtgtcttttctggaatttggtaccgaggattgaagagcacggttatc	
pKan_L3S1P22-F	aaggccgcaaatcgcggcctttttttattgataacaaaagtatgaagagcgggacag	pKan-Ter7
pKan_L3S1P22-R	taaaaaaggccgcgatttgcggccttattgttcgtcgattgaagagcacggttatc	
pKan_L3S2P44-F	ttattgaagacgctgaaaagcgtcttttttttgtttcggtccgtatgaagagcgggacag	pKan-Ter8
pKan_L3S2P44-R	acgcttttcagcgtcttcaataattggtttggtaccgaggattgaagagcacggttatc	
pKan_L3S2P42-F	aataaaaagacgctgaaaagcgtctttttatttttcggtccgtatgaagagcgggacag	pKan-Ter9
pKan_L3S2P42-R	acgcttttcagcgtctttttatttttctttggtaccgaggattgaagagcacggttatc	
pKan_L3S1P00-F	ggggagcgggaaaccgctccccttttttattgataacaaaagtatgaagagcgggacag	pKan-Ter10
pKan_L3S1P00-R	aaaaaggggagcggtttcccgctccccttattgttcgtcgattgaagagcacggttatc	
pKan-F	tgaagagcgggacagtaagacgggtaag	pKan backbone
pKan-R	tgaagagcacggttatccacagaatcag	
CymR-F	tgtggataaccgtgctcttcaatgatcatgtcccccaagcgg	pKan-CymR
CymR-R	tcttactgtcccgctcttcactatcaccgcttgaacttggcgtacc	
sfGFP-F	gtggataaccgtgctcttcaatgcgtaaaggcgaggagctgttcactggtgtc	pKan-sfGFP
sfGFP-R	tcttactgtcccgctcttcactatcatttgtacagttcatcca	
mCherry-F	gccgctgcagaaagaggagaaatactagatggtcagcaagggcgaggaggacaaca	pKan-mCherry
mCherry-R	tcttactgtcccgctcttcactattacttgtacagctcgtccatg	
SP11-F1	gtggataaccgtgctcttcagtatgttcacattcgaaccgtctctgctttgaca	pKan-SP11-RBS14
SP11-F2	gaaccgtctctgctttgacaatggcttgaattggggtgtaaagtcgtggcca	
SP11-R3	tcttactgtcccgctcttcacatgaaccgatctcctcgttaggtggccacgactttacacc	
SP30-F2	gaaccgtctctgctttgacatcgtgtggcgcttgggtgtaaagtcgtggcca	pKan-SP30-RBS14
SP11-CuO-P1	ataatacaaacagaccagattgtctgtttgtttggccacgactttacacc	pKan-SP11-CuO-RBS15
SP11-CuO-P2	tcttactgtcccgctcttcacatatggacactccttacttagaataatacaaacagaccagattg	pKan-SP30-CuO-RBS15
**Protospacers for CUBIC plasmids**		
sfGFP-F	gccacatccagttccaccagaata	pCB1-sfGFP
sfGFP-R	aaactattctggtggaactggatg	
SCO2082-F	gccacgatgactttgatgactgcg	pCB1-ftsZ$_{SC}$
SCO2082-R	aaaccgcagtcatcaaagtcatcg	
SCO1541-F	gccacgtggtgttcataagcgcca	pCB1-ssgB$_{SC}$
SCO1541-R	aaactggcgcttatgaacaccacg	
SCO1950-F	gccagacctccgcctttctgcagc	pCB1-whiA$_{SC}$
SCO1950-R	aaacgctgcagaaaggcggaggtc	
SCO5877-F	gccagttctccgcactcccatgag	pCB1-redD$_{SC}$
SCO5877-R	aaacctcatgggagtgcggagaac	
SCO5085-F	gccaatcccgcatcggtgattaca	pCB1-actII-orf4$_{SC}$
SCO5085-R	aaactgtaatcaccgatgcgggat	

DivIVA$_{SC}$-F	gccacctcgtcatagccttctcgg	pCB1-divIVA$_{SC}$
DivIVA$_{SC}$-R	aaacccgagaaggctatgacgagg	
DivIVA$_{SV}$-F	gccacttgttccgcacgtcctcgg	pCB1-divIVA$_{SV}$
DivIVA$_{SV}$-R	aaacccgaggacgtgcggaacaag	
DivIVA$_{SRS}$-F	gccacctcgtcatagccttctcgg	pCB1-divIVA$_{SRS}$
DivIVA$_{SRS}$-R	aaacccgagaaggctatgacgagg	
DivIVA$_{SRF}$-F	gccactcgttctcgcggagcagac	pCB1-divIVA$_{SRF}$
DivIVA$_{SRF}$-R	aaacgtctgctccgcgagaacgag	
DnaA$_{SC}$-F	gccaagaagttgctccaatacgcg	pCB1-dnaA$_{SC}$
DnaA$_{SC}$-R	aaaccgcgtattggagcaacttct	
DnaA$_{SV}$-F	gccagaggacgcgtggccacactg	pCB1-dnaA$_{SV}$
DnaA$_{SV}$-R	aaaccagtgtggccacgcgtcctc	
DnaA$_{SRS}$-F	gccacagcactcgcggccacactg	pCB1-dnaA$_{SRS}$
DnaA$_{SRS}$-R	aaaccagtgtggccgcgagtgctg	
DnaA$_{SRF}$-F	gccacgtccttggactcgacgccc	pCB1-dnaA$_{SRF}$
DnaA$_{SRF}$-R	aaacgggcgtcgagtccaaggacg	
Check-f	aaccgcttcgtcgtggagga	CUBIC validation
Check-r	cttcttgatcgagtgccggt	

Table S5. Cost summary for making CUBIC constructs.

	Price (€)	Quantity	Sum (€)	Source
Oligonucleotide_F	0.1 / nt	24 nt	2.4	IDT
Oligonucleotide_R	0.1 / nt	24 nt	2.4	IDT
T4 PNK	0.096 / unit	1 unit	0.096	New England BioLabs
BsaI	0.063 / unit	2 units	0.126	New England BioLabs
T4 Ligase	0.0028 / unit	40 units	0.1152	New England BioLabs
T5 Exonuclease	0.0588 / unit	5 units	0.294	New England BioLabs
Total (€)	5.4 € per CUBIC plasmid			

Supplementary References

(1) Kieser, T.; Bibb, M. J.; Buttner, M. J.; Chater, K. F.; Hopwood, D. A. *Practical Streptomyces Genetics*; John Innes Foundation Norwich, 2000.
(2) Bai, C.; Zhang, Y.; Zhao, X.; Hu, Y.; Xiang, S.; Miao, J.; Lou, C.; Zhang, L. Exploiting a precise design of universal synthetic modular regulatory elements to unlock the microbial natural products in *Streptomyces*. *Proc. Natl. Acad. Sci. U.S.A.* **2015**, *112* (39), 12181-12186.
(3) Chen, Y.-J.; Liu, P.; Nielsen, A. A.; Brophy, J. A.; Clancy, K.; Peterson, T.; Voigt, C. A. Characterization of 582 natural and synthetic terminators and quantification of their design constraints. *Nat. Methods* **2013**, *10* (7), 659-664.
(4) Zhang, S.; Zhao, X.; Tao, Y.; Lou, C. A novel approach for metabolic pathway optimization: Oligo-linker mediated assembly (OLMA) method. *J. Biol. Eng.* **2015**, *9* (1), 1-10.
(5) Zhao, X.; Wei, W.; Zong, Y.; Bai, C.; Guo, X.; Zhu, H.; Lou, C. Novel switchable ECF sigma factor transcription system for improving thaxtomin A production in *Streptomyces*. *Synth. Syst. Biotechnol.* **2022**, *7* (3), 972-981.
(6) Floriano, B.; Bibb, M. *afsR* is a pleiotropic but conditionally required regulatory gene for antibiotic production in *Streptomyces coelicolor* A3 (2). *Mol. Microbiol.* **1996**, *21* (2), 385-396.
(7) Willemse, J.; Mommaas, A. M.; van Wezel, G. P. Constitutive expression of *ftsZ* overrides the *whi* developmental genes to initiate sporulation of *Streptomyces coelicolor*. *Antonie Leeuwenhoek* **2012**, *101*, 619-632.
(8) Van der Aart, L. T.; Nouioui, I.; Kloosterman, A.; Igual, J. M.; Willemse, J.; Goodfellow, M.; Van Wezel, G. P. Polyphasic classification of the gifted natural product producer *Streptomyces roseifaciens* sp. nov. *Int. J. Syst. Evol.* **2019**, *69* (4), 899-908.
(9) Gentz, R.; Langner, A.; Chang, A. C.; Cohen, S. N.; Bujard, H. Cloning and analysis of strong promoters is made possible by the downstream placement of a RNA termination signal. *Proc. Natl. Acad. Sci. U.S.A.* **1981**, *78* (8), 4936-4940.
